# Pancreatic follicular dendritic cell sarcoma: a case report

**DOI:** 10.1186/s12957-023-03213-4

**Published:** 2023-10-13

**Authors:** Jing Lou, Runyu Xia, Guoli Li, Jun Su, Hong Zheng

**Affiliations:** Department of Pathology, The Affiliated Hospital of Zunyi Medical University, Zunyi City, Guizhou Province People’s Republic of China

**Keywords:** Pancreas, FDCS, Pathological features, Differential diagnosis

## Abstract

**Background:**

Follicular dendritic cell sarcoma (FDCS) is a rare, low-to-moderate-grade malignant tumor, which occurs in the dendritic cells of the germinal center. Pancreatic FDCS (PFDCS) is extremely rare, with only a few reported cases. Presently, the etiology and pathogenesis of pancreatic FDCS are still unclear, and the clinical symptoms and signs as well as the laboratory diagnosis lack specificity. Although PFDCS presents better histological and morphological characteristics and a distinct immunophenotype, it can be easily missed and/or misdiagnosed if it occurs outside the node. Lymph node FDCS are easier to diagnose because of the rarity of fusiform cell tumors in lymph nodes.

**Case demonstration:**

Herein, we reported a 67-year-old female patient with upper-left abdominal pain without obvious cause and was admitted for treatment. A computed tomography (CT) scan revealed a cystic solid mass in the pancreatic tail toward the greater curvature of the stomach, with an obvious enhancement of the cyst wall in enhanced scanning. Subsequently, the patient underwent surgical resection and the resected sample was sent for pathological biopsy. According to the results, the pathology was consistent with the histological morphology and immunohistochemical characteristics of FDCS, and the Epstein–Barr virus (EBV)-encoded RNA was negative for in situ hybridization. Three months post-resection, the patient returned to the hospital for chemotherapy. This case report is aimed to improve the clinical recognition of FDCS.

**Conclusion:**

Pancreatic FDCS is a rare disease. Herein, we have reported a case of pancreatic FDCS and analyzed its clinical and pathological features and differential diagnosis to improve the understanding of FDCS.

## Background

Follicular dendritic cell sarcoma (FDCS) is a rare and inert tumor, which accounts for approximately 0.4% of all soft-tissue sarcomas. It can occur in any body part, with approximately 1/2–2/3 of occurrence in the lymph nodes and remaining in the other organs outside the lymph nodes. The most common extranodal location is the abdominal cavity and pelvic region, followed by the neck and chest, and rarely involves soft tissues, including the breast, thigh, groin, dura mater, and skin [1]. Because the disease is invasive, prone to metastasis and recurrence, exhibits a poor prognosis, and lacks typical clinical phenotypes and imaging features, early diagnosis and treatment are crucial for patients with FDCS. Therefore, it is important to recognize the pathological manifestations of FDCS.

## Case demonstration

**Fig. 1:**
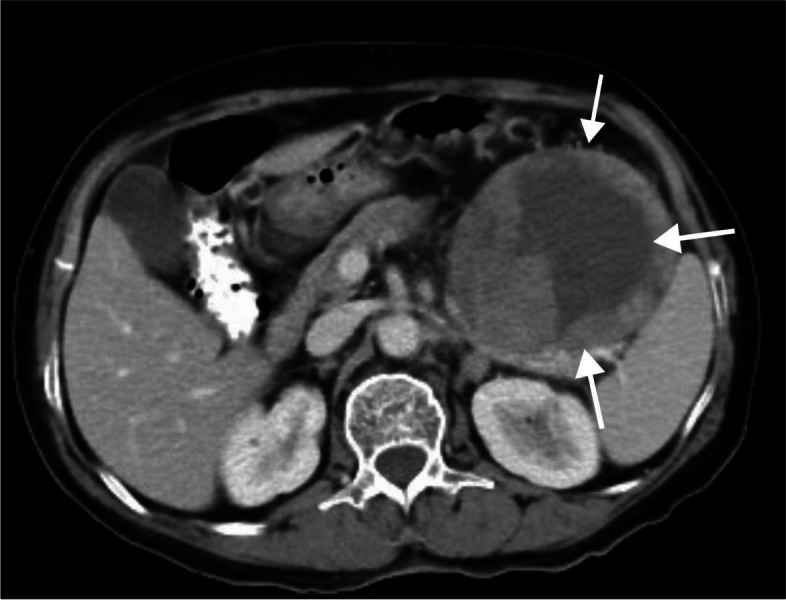
A cystic solid mass in the caudal region of the pancreas toward the greater curvature of the stomach, with unclear boundaries with the surrounding gastric wall and intestines (white arrows)

The case was of a 67-year-old female patient who presented with intermittent pain in the upper-left abdomen without any obvious inducement. The patient had a history of grade 3 essential hypertension and belonged to a very high-risk group. The patient regularly took antihypertensive drugs for blood pressure control. During the physical examination, a mass of approximately 5 cm was found and could be palpated by palpation in the lower-left abdomen, which was hard and inactive. Laboratory tests showed alkaline phosphatase 152 U/L, glutamyl transferase 107 U/L, and β2 microglobulin 3030.00 ng/ml; other blood lipids, blood glucose, renal function, gastrointestinal tumor markers (CA199, CA125, and CEA), blood routine, and urine routine were normal. Abdominal CT (Fig. 1) showed a cystic solid mass in the pancreatic tail, toward the greater curvature of the stomach, which had unclear boundaries and was surrounded by the gastric wall and intestines. The solid part in the enhanced scan was considerably enhanced, and the circular cystic mass was primarily seen in the area near the bottom of the pancreas, with the stomach above it. Multiple tortuous and enhanced vascular shadows were observed near the bottom of the stomach. The oval cystic mass was seen in the pancreatic tail, with an obvious enhancement in the quality of the cystic wall during scanning. Preliminary consideration of gastrointestinal stromal tumor (GIST) or pancreatic neuroendocrine tumor (PNENs). In order to determine the nature of the tumor, a needle biopsy was performed on the pancreatic mass, and the pathological results showed malignant tumor, which was considered as myeloid sarcoma, but no abnormalities were found in the bone marrow examination. Subsequently, after multidisciplinary consultation, surgical resection of “open pancreatic body and tail + total splenectomy” was proposed.

**Fig. 2:**
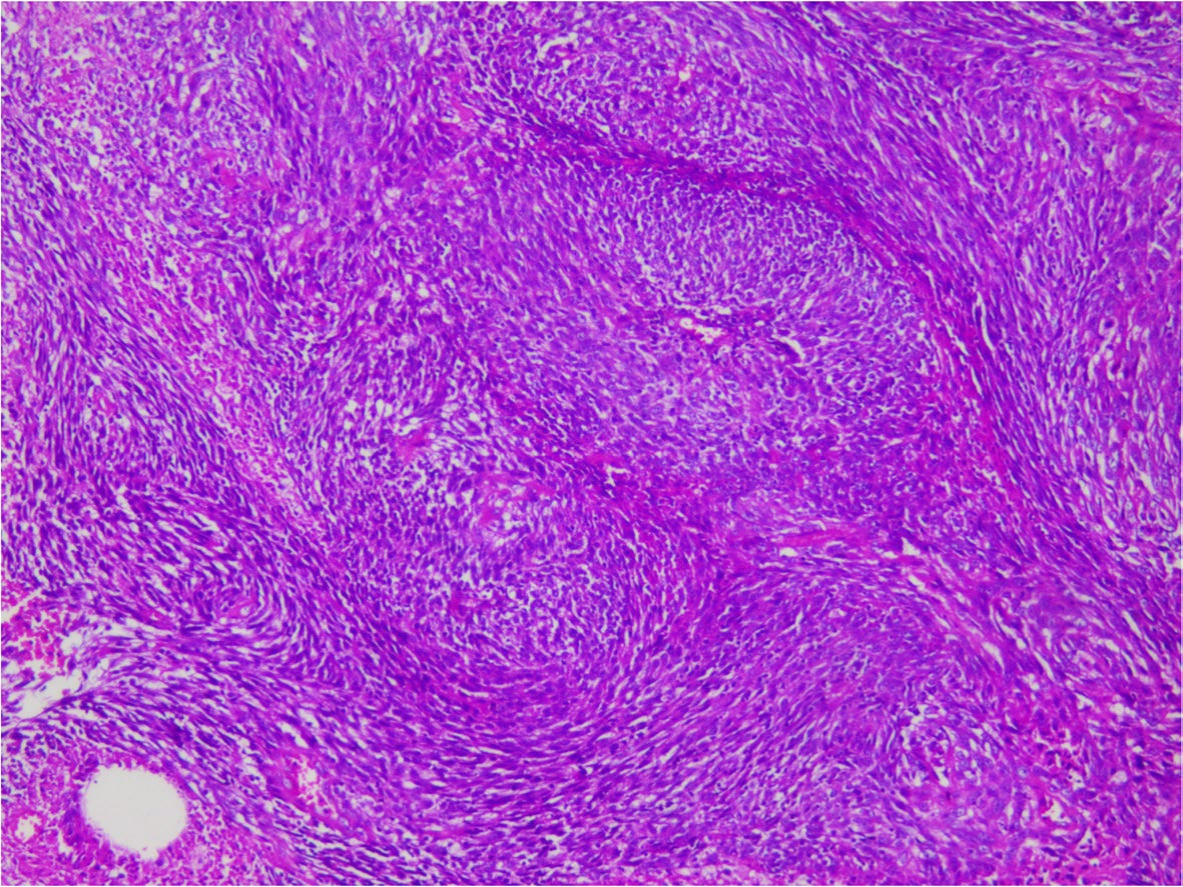
Low-magnification images of tumor cell arrangement in a woven and swirling pattern, similar to meningioma (× 200)

**Fig. 3:**
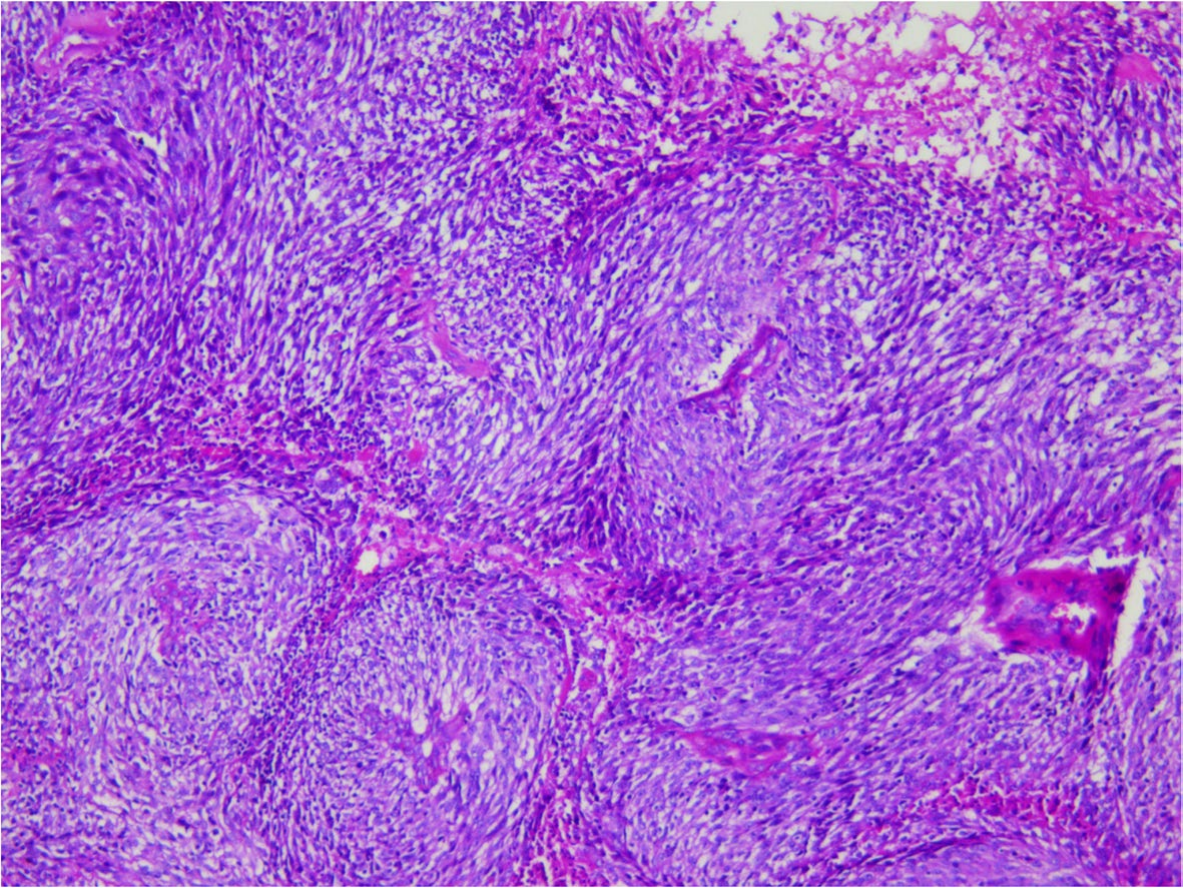
Tumor cells arrangement in a concentric circle around blood vessels, with visible localized necrosis (× 200)

**Fig. 4:**
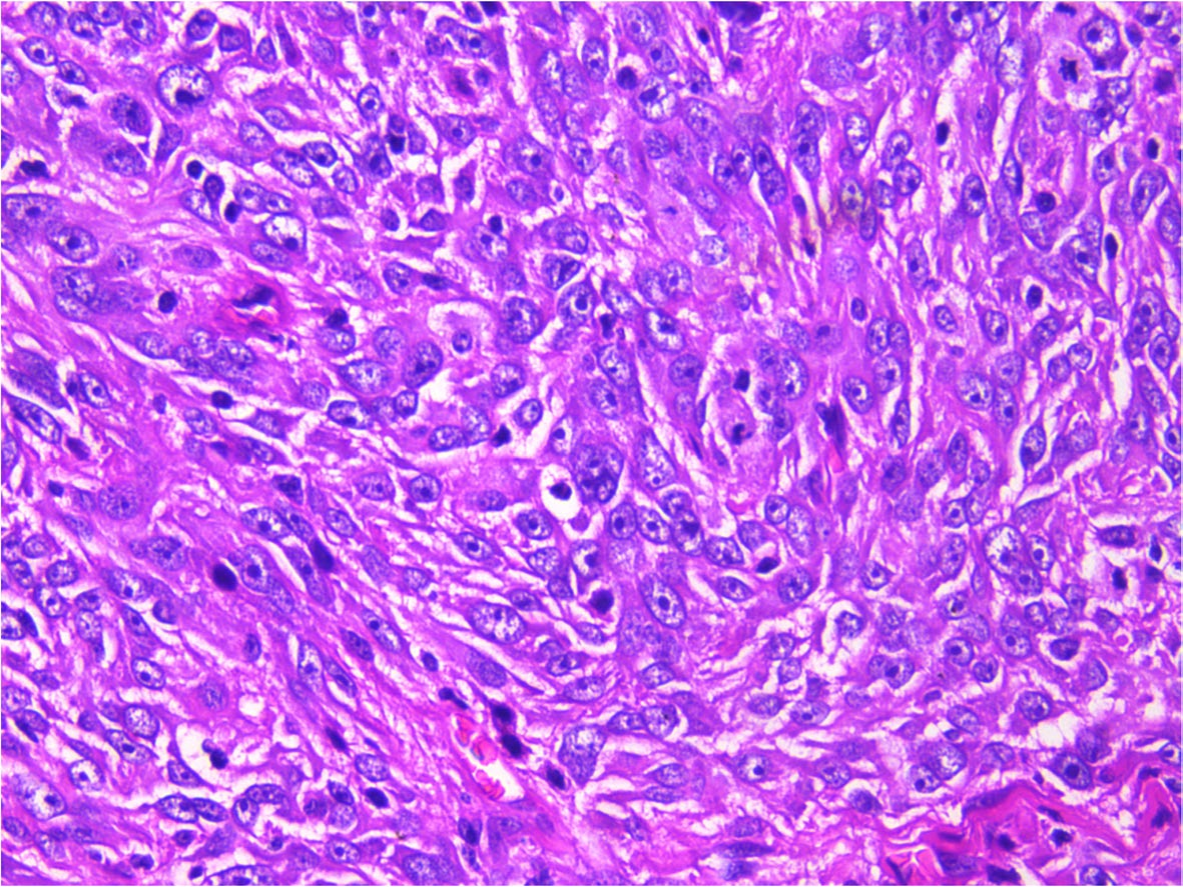
High-magnification images of tumor cell shapes, spindle, circular, or oval shapes, with unclear boundaries with the surrounding area. The nuclei are large and deeply stained, with mild anisotropy. The cytoplasm is lightly stained and eosinophilic, with visible mitotic structures (× 400)

**Fig. 5:**
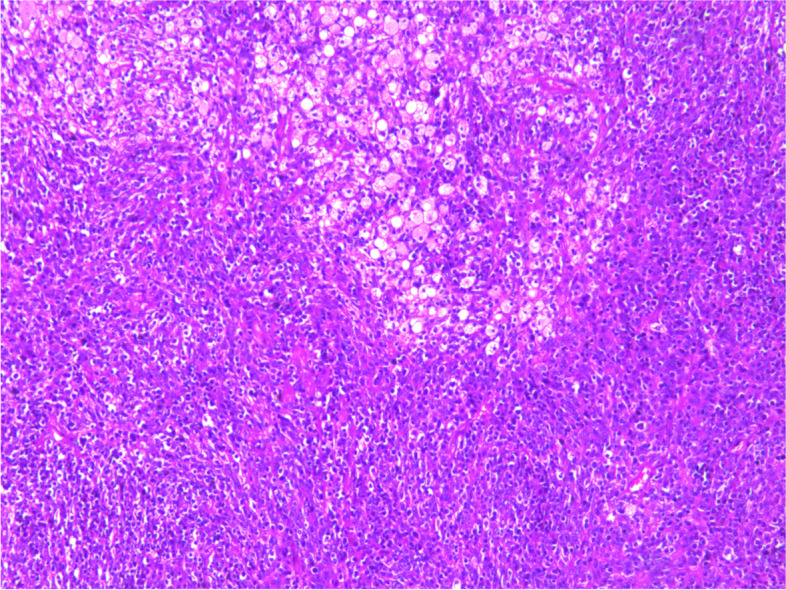
Vacuole-like cells (× 200)

**Fig. 6:**
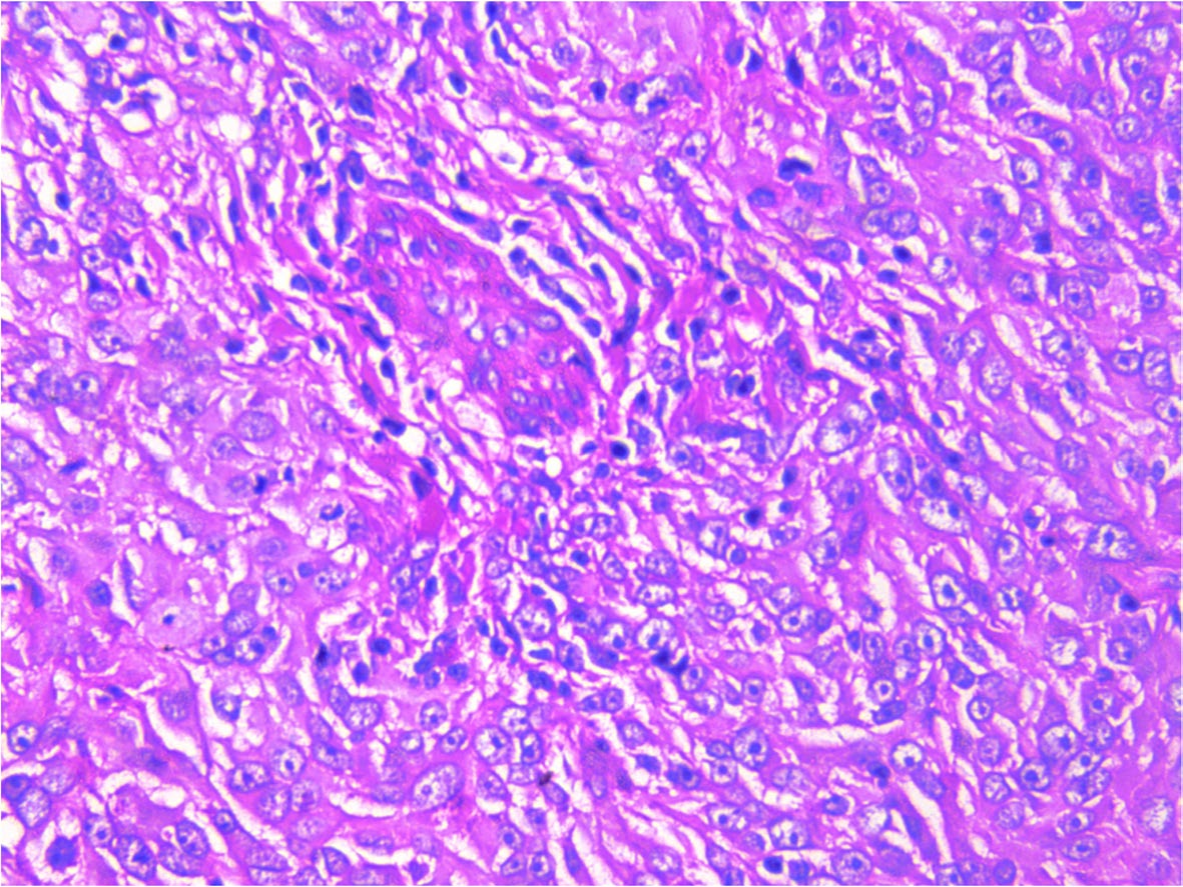
Polynuclear tumor cells (× 400)

**Fig. 7:**
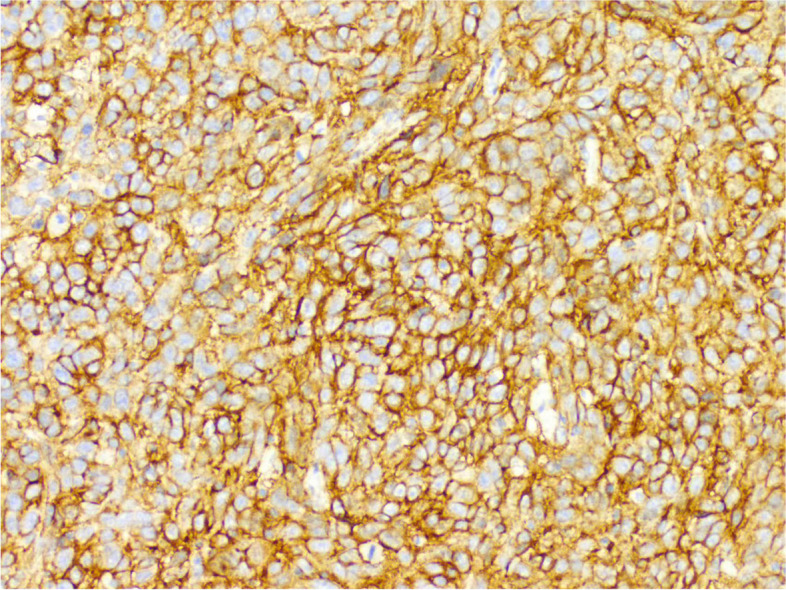
Immunohistochemistry showing CD21 + (× 400)

**Fig. 8:**
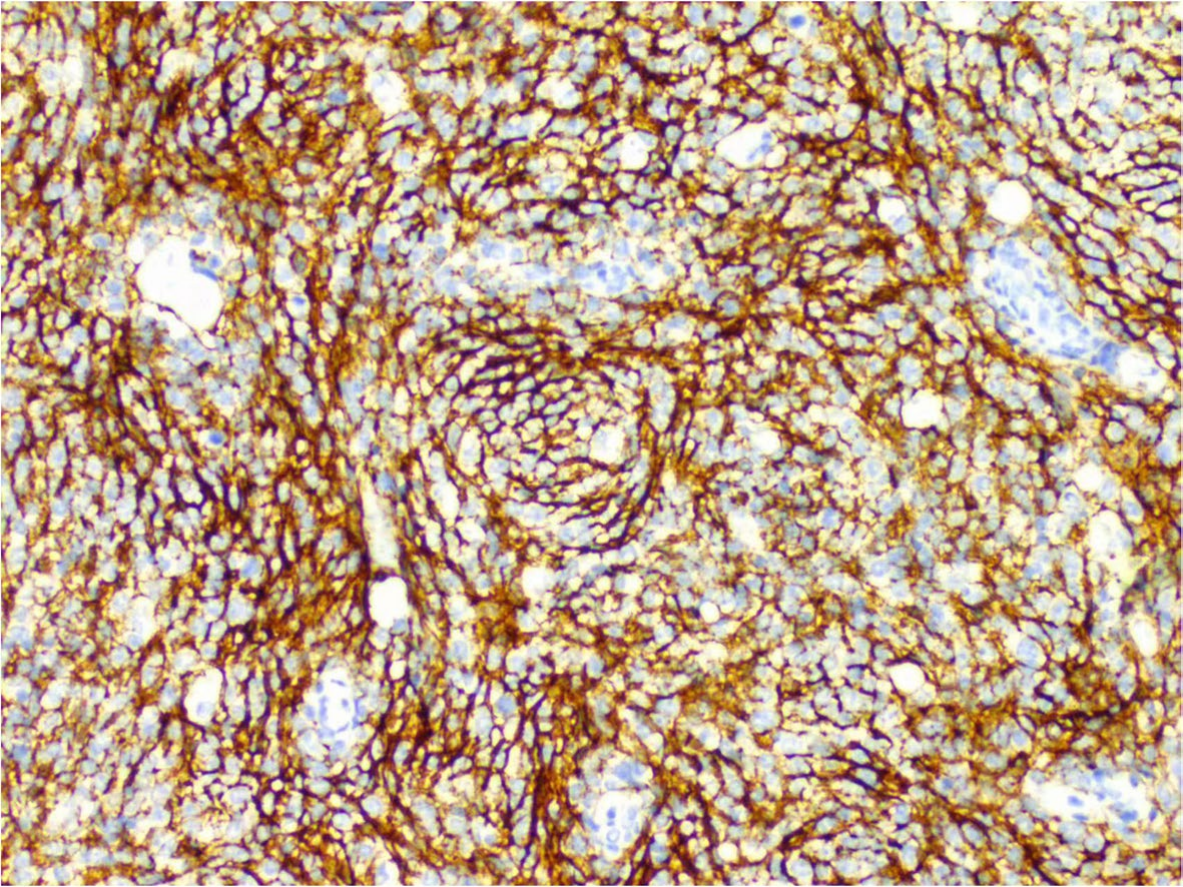
Immunohistochemistry showing CD35 + (× 400)

**Fig. 9:**
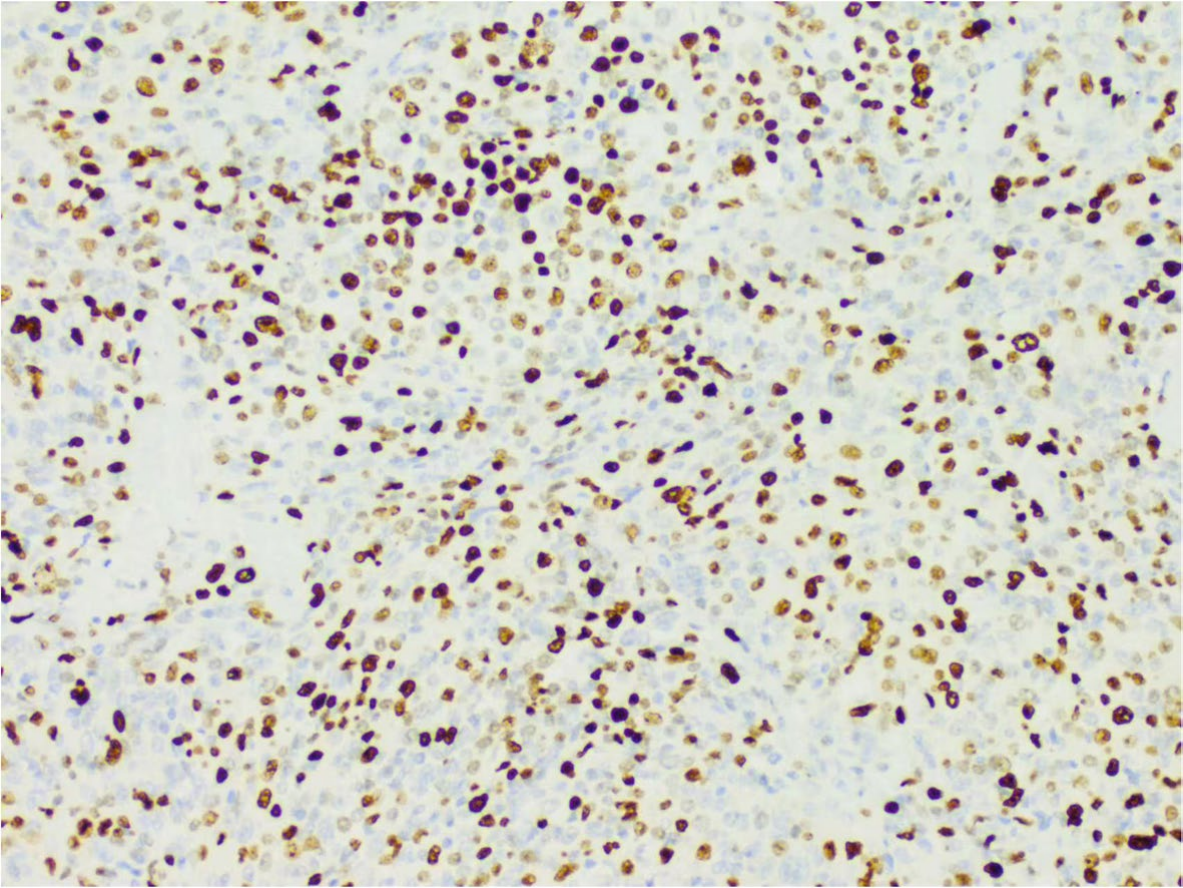
Immunohistochemistry showing Ki-67 + (× 400)

During the operation, a mass was found close to the splenic hilum, with a size of about 120 × 120 × 100 mm, which adhered to the surrounding tissues; after exploration of the pancreas, a mass was found in the tail of the pancreas, with a size of about 60 × 50 mm. It was tightly adhered to the gastric wall tissue, and the splenic artery and vein were wrapped in it, which was difficult to separate. When the mass was separated, it was found that the boundary between the mass and the spleen was not clear, and the mass at the tail of the pancreas was adhered to, and the mass at the tail of the pancreas was densely adhered to the stomach wall. The splenic hilar mass was completely separated, and the boundary between it and the mass in the tail of the pancreas was unclear. The spleen and the mass in the tail of the pancreas were further separated until it was free and the spleen and part of the pancreas were removed. A total of two tumors were found in the postoperative gross specimen, with a mass size of approximately 95 × 76 × 70 mm and located close to the splenic hilum. The cut surface was soft and grayish-white and grayish-yellow in color. Necrosis was seen in some areas, which looked like fish meat. The surface envelope of the tumors was complete. The pancreatic tail was approximately 55 × 43 × 40 mm in size, with parts of the envelope attached. At low magnification, tumor cells seemed to be arranged in a braided and whirlpool pattern (Fig. 2), similar to meningiomas, and in a concentric circle around the blood vessels, with visible necrosis in some regions (Fig. 3). At high magnification, tumor cells were visible as spindle-, circular-, or oval-shaped, with unclear boundaries with the surrounding area. The nuclei were large and deeply stained, with mild anisotropy. The cytoplasm was lightly stained and eosinophilic, with visible mitotic structures (> 5/10 HPF) (Fig. 4), visible vacuole-like cells (Fig. 5), and a few multinucleated tumor cells (Fig. 6). Additionally, a few lymphocytes and plasma cells were scattered among tumor cells. Immunohistochemical analysis showed the following characteristics of the tumor cells: cytokine (CK, −), cluster of differentiation (CD) 45 (weak +), CD20 ( −), CD3 ( −), CD5 ( −), CD34 (partial tumor cells +), CD43 (partially weak +), CD117 ( −), myeloperoxidase (MPO, +), e-cadherin ( −), CD10 ( −), CD15 ( −), CD163 ( −), CD235a ( −), CD61 ( −), CD138 ( −), anaplastic lymphoma kinase ( −), CD21 ( +) (Fig. 7), CD23 ( −), CD35 ( +) (Fig. 8), CD68 ( −), langerin ( −), S-100 ( −), smooth muscle actin (SMA, −), and Ki-67 (approximately 50% +) (Fig. 9). Furthermore, we performed in situ hybridization (ISH) for EBV-encoded RNA and found that EBV region/ISH was negative.

The patient returned to the hospital for chemotherapy treatment 3 months after resection. CT re-examination was performed during the chemotherapy, revealing liver and retroperitoneal lymph node metastasis. Thereafter, the CHOP (cyclophosphamide + doxorubicin + vincristine + dexamethasone) regimen was given for chemotherapy. After discharge, prednisone acetate was taken orally by the patient for 3 days, and regular chemotherapy was practiced. The patient was followed up for 13 months and did not relapse; the general condition is fine.

## Discussion

FDCS was first reported by Monda et al. [2] in 1986. In 2016, the World Health Organization classified histiocytic tumors and dendritic cell tumors into one category. FDCS origins from follicular dendritic cells in the lymphatic tissues and is a very rare low-grade malignant tumor. Wang et al. [3] reported that FDCS was prone to recurrence and should be considered an intermediate-grade malignant tumor, especially in the case of intraperitoneal FDCS. To date, only seven cases of pancreatic FDCS have been reported in the literature (Table 1). Herein, we reported a single case of pancreatic FDCS, analyzed its clinical and pathological features, and differential diagnoses to improve the understanding of the pathogenesis and characteristics of pancreatic FDCS.

**Table 1:** Reported clinical characteristics of follicular dendritic cell sarcoma of the primary pancreas

References	Year of publication	Age (Y)	Sex	Tumor diameter (cm)	Location	Presented symptoms	EBER/ISH	Metastasis
Hollowood et al. [4]	1995	63	M	15	Head	An epigastric mass, nausea, and weight loss	/	None
Shen et al. [5]	2006	64	M	10.5	Head	Weight loss, poor appetite, and abdominal fullness	Negative	Liver
Liang et al. [6]	2016	67	F	4	Tail	The patient presented with no symptoms	Negative	None
Soriano et al. [7]	2007	56	M	2	**/**	Chest discomfort	/	Liver and abdominal lymph node
Kang et al. [8]	2018	25	F	7.2	Head	Nausea and abdominal discomfort	/	Liver, pancreas, and retroperitoneal lymph nodes
Lu et al. [9]	2019	49	F	5	Tail	Repeated ptosis of both eyelids and oral ulcers and erosions	Negative	None
Lu et al. [10]	2022	30	M	10	Head	Jaundice, abdominal fullness, and weight loss	Negative	Liver
Our case	2022	67	F	5.5	Tail	Pain in the left upper abdomen	Negative	Liver

*M* Male, *F* Female, *EBER* Epstein–Barr encoding region, *ISH* In situ hybridization

The onset age of pancreatic FDCS is between 25 and 67 years of age, with a wide age distribution and a male-to-female ratio of 1:1, with an insignificant difference in the male-to-female ratio. To date, the etiology and pathogenesis of pancreatic FDCS are still unclear, with no clear etiology for most cases. FDCs originate from interstitial cells and are associated with B cells in lymphoid follicles. They primarily transmit processed antigens to B lymphocytes and stimulate B cell proliferation and differentiation. Although they are involved in maintaining the lymphoid follicular environment and activating B cells in the lymphoid follicles, no antigen presentation or endocytosis is found [11]. Soriano et al. [7] reported that approximately 20% of FDCS was accompanied by Castleman disease (CD), suggesting a correlation between FDCS and CD occurrences. Some researchers believed that transparent vascular CD might induce precancerous lesions of FDCS. Starr et al. [12] reported that a patient with thyroid FDCS had mutations in the following three genes: *PTEN*, *RET*, and *TP53*. Griffin et al. [13] also detected the loss of function of tumor suppressor genes regulating nuclear factor-κB and cell cycle pathways during their study on FDCS. Moreover, according to previous studies, approximately 12% of FDCS cases have been correlated with EBV [5]. The correlation between FDCS and EBV varies among different organs, with almost all liver and spleen FDCS being correlated with EBV [14]. According to previous studies, both patients with pancreatic FDCS and our patients did not have an EBV infection.

FDCS is often manifested as a painless mass with atypical clinical symptoms. According to studies, the clinical manifestations of pancreatic FDCS include weight loss, abdominal pain, abdominal masses, nausea, anorexia, and abdominal distension. Pancreatic FDCS can metastasize to abdominal and retroperitoneal lymph nodes through lymph nodes and can also spread to the liver through hematogenous spread. Imaging presents pancreatic FDCS as an exogenous and well-defined solid or cystic solid mass with a rich blood supply, showing solid and cystic wall enhancement under enhanced scanning [6, 8].

The diagnosis of pancreatic FDCS mainly depends on pathology and is based on relatively specific histological manifestations and specific immunohistochemical phenotypes, which were as follows: (1) microscopic view, circular, spindle-shaped, and epithelioid tumor cells with woven, vortex-shaped, and mat-like structures, similar to meningioma; (2) unclear tumor cell boundaries, with fibrous septa arranged in concentric circles around blood vessels; (3) large and deeply stained nuclei and lightly stained and eosinophilic cytoplasm, with slightly heterogeneous nucleus showing visible mitotic structures (> 5/10 HPF); (4) visible vacuole-like cells and a few multinucleated tumor cells, with visible local necrosis; and (5) a few lymphocytes and plasma cells scattered among tumor cells. The characteristic immunophenotypes for diagnosing FDCS are the CD21, CD23, and CD35 markers of FDCs, with varying degrees of CD45, CD68, and S-100 levels. T and B lymphocyte markers are negative, whereas CK, SMA, and langerin are usually negative. CD163 is negative in dendritic cell tumors. In this case, CD21 and CD35 were positive and CD45 was weakly positive; T and B cell markers were negative; MPO was positive, but CD117, CD15, and CD68 were negative; therefore, myeloid sarcoma and acute myeloid leukemia were not considered. Negative CK excluded tumors of epithelial origin and SMA ( −) excluded meningiomas. S-100 and langerin were negative; therefore, Langerhans cell histiocytosis was not considered. Therefore, the final diagnosis was FDCS. FDCS needs to be differentiated from the following tumors: (1) myeloid sarcoma: common sites include skin, lymph nodes, and bones; the tumor cells infiltrate and grow with unclear boundaries, with a diffused, nested, and patchy arrangement; the tumor cells are mostly round and ovoid shapes with less amount of cytoplasm; visible kidney-shaped or lobulated deeply stained nucleus; immunohistochemistry shows MPO, lysozyme, CD68, and CD43 expression, with CD43 and lysozyme being most sensitive markers, whereas CD21, CD23, and CD35 are not expressed; (2) acute myeloid leukemia: a common malignant tumor in the hematological system that primarily diagnosed based on laboratory examinations and bone marrow puncture; tumor cells express CD45, MPO, CD117, CD15, CD68, and CD43, with a small portion expressing CD34 and CD56; (3) pleomorphic undifferentiated sarcoma: also known as malignant fibrous histiocytoma; mainly occurs in the soft tissues of limbs and trunk; the tumor cells have notable pleomorphism and are arranged in a striped or spoke shape; the tumor cell nucleus is deeply stained, the cytoplasm is rich, and easily visible mitotic stage; immunohistochemical analysis shows that CD68 and lysosome enzymes have positive expression, whereas that of CD21, CD23, and CD35 is negative; (4) spindle cell carcinoma: prominent spindle-shaped cells arranged in sheets, bundles, and nests; cells usually express broad-spectrum proteins (CKs), but do not express CD21 or CD35; (5) ectopic meningioma: extremely similar morphology to FDCS, with spindle-shaped cells arranged in a spiral pattern; however, tumor cells express SMA and not the FDCS-related antigens; (6) Langerhans cell histiocytosis: tumor cells have an increased size, rich cytoplasm, obvious nucleolus, nuclear groove, and infiltration of eosinophilic granulocytes and other inflammatory cells; immunohistochemistry shows positive expression of CD1a, langerin, and S-100 proteins; and (7) interdigitating dendritic cell sarcoma: a rare tumor with extranodal lesions occurring in the snuff, tonsils, liver, spleen, gastrointestinal tract, and breasts; it is clinically invasive and has a pathological morphology similar to FDCS; immunohistochemistry shows S-100 ( +) cells and positive expression of CD45 and CD68 to varying degrees; however, CD21 and CD35, and CD1a or langerin are not expressed.

To date, no standard treatment method is available for pancreatic FDCS, and study has shown that some patients with FDCS exhibit partial reactions after two cycles of chemotherapy with the CHOP regimen [15]. However, postoperative radiotherapy does not seem to improve the progression-free survival and overall survival of patients nor does it affect their recurrence rate. Therefore, radical resection is still the main treatment for pancreatic FDCS, and the value of radiotherapy and chemotherapy for the treatment of this tumor is uncertain.

In conclusion, FDCS of the pancreas is a rare and easily misdiagnosed malignant tumor, which lacks typical clinical symptoms, signs and laboratory tests, and its diagnosis is challenging. Improving the understanding of the morphology and immunohistochemistry of FDCS is helpful for the accuracy of diagnosis. The number of cases in the pancreas is small, and our late follow-up and observation of this tumor are limited, so we need to further explore the best treatment for this tumor.

## Data Availability

All the data regarding the findings are available within the manuscript.

## Abbreviations

CT: Computed tomography
FDCS: Follicular dendritic cell sarcoma
FDC: Follicular dendritic cell
CK: Cytokine
CD: Cluster of differentiation
MPO: Myeloperoxidase
SMA: Smooth muscle actin
ISH: In situ hybridization
EBV: Epstein–Barr virus
EBER: Epstein–Barr encoding region
CD: Castleman disease
